# Supplementary material for: Risk Factors of Multiple Primary Cancers Among Colorectal Cancer Survivors
Source: Cancers (Basel). 2025 Jun 25;17(13):2145. doi: 10.3390/cancers17132145 (PMC12249427; doi:10.3390/cancers17132145)
Supplement: Supplementary file 1 [file cancers-17-02145-s001.zip › cancers-3693224-supplementary.pdf]

Supplementary files

A. Supplementary Tables

Supplementary Table S1: Univariable and multivariable competing risk regression analysis of MPC with sociodemographic, behavioural, and clinical factors among individuals diagnosed with index CRC at Flinders Medical Centre, excluding cases with MMR protein loss who did not undergo genetic testing but with no indication of and/or known family history of lynch syndrome (n=538).

Variables	Categories	Univariable analysis		Multivariable analysis	
		SHR (95%CI)	P-value	SHR (95%CI)	P-value
Age at CRC diagnosis, year	<65	1.0		1.0	
	>=65	3.47 (1.92, 6.27)	<0.001	2.94 (1.50, 5.76)	0.002
Sex	Male	1.63 (0.92, 2.87)	0.094	1.90 (1.06, 3.38)	0.031
	Female	1.0			
Site of index CRC	Colon	1.75 (0.97, 3.17)	0.065	0.82 (0.33, 2.02)	0.663
	Rectum	1.0		1.0	
Stage of Index CRC at diagnosis	Stage I and II	2.37 (1.37, 4.10)	0.002	2.01 (1.08, 3.72)	0.027
	Stage III and IV	1.0		1.0	
Expression of MMR protein(s)	Reduced	2.70 (1.15, 6.32)	0.022	2.04 (0.98, 4.61)	0.051
	Normal	1.0		1.0	
Surgery for index CRC	Yes	1.96 (1.01, 3.81)	0.047	0.86 (0.32, 2.31)	0.761
	No	1.0		1.0	
Chemotherapy for Index CRC	Yes	1.0		1.0	
	No	2.52 (1.33, 3.82)	0.003	0.96 (0.50, 1.81)	0.888
Radiotherapy for Index CRC	Yes	1.0		1.0	
	No	2.83(1.29, 6.22)	0.010	2.66 (0.71, 10.0)	0.148
Hypertension	Yes	1.73 (1.03, 2.88)	0.037	1.03 (0.58, 1.86)	0.912
	No	1.0		1.0	

CRC: colorectal cancer; CI: confidence interval; MMR: mismatch repair; SHR: sub-distribution hazard ratio

Supplementary Table S2: Univariable competing risk regression analysis of MPC with sociodemographic, behavioural and clinical factors among individuals diagnosed with index CRC at Flinders Medical Centre

Characteristics		SHR (95%CI)	P-value
Age at the diagnosis of index CRC, years	<65	1.0	
	≥65	3.61 (2.00, 6.49)	<0.001
Sex	Female	1.0	
	Male	1.44 (0.85, 2.45)	0.181
Socioeconomic status(quintile)	Lowest to low	1.0	
	Middle to highest	1.32(0.80, 2.16)	0.276
Smoking habit	No smoking	1.0	
	Current/previous smoker	1.10 (0.67, 1.80)	0.707
Alcohol drinking habit	Less risky or no alcohol consumption	1.0	
	Risky alcohol consumption	1.43 (0.81, 2.52)	0.223
Site of index CRC	Rectum	1.0	
	Colon	1.82 (1.01, 3.28)	0.047
Tumour differentiation	Moderate to well-differentiated	1.0	
	Poorly differentiated	1.11 (0.60, 2.05)	0.742
Stage of CRC at diagnosis	Stage III and IV	1.0	
	Stage I and II	2.34 (1.37, 3.99)	0.002
MMR protein(s) expression	Normal	1.0	
	Loss	2.47 (1.23, 4.96)	0.011
Surgery for index CRC	No	1.0	
	Yes	2.03 (1.05, 3.93)	0.036
Chemotherapy	Yes	1.0	
	No	2.07 (1.24, 3.45)	0.005
Radiotherapy	Yes	1.0	
	No	2.52 (1.20, 5.30)	0.014
BMI	<25kg/m^2	1.0	
	≥25kg/m^2	1.04 (0.60, 1.81)	0.901
Hypertension	No	1.0	
	Yes	1.75 (1.06, 2.89)	0.028
Cardiac diseases	No	1.0	
	Yes	1.38 (0.83, 2.29)	0.214
Diabetes Mellitus	No	1.0	
	Yes	1.41 (0.79, 2.51)	0.248
Chronic respiratory disease	No	1.0	
	Yes	1.32 (0.76, 2.26)	0.323

BMI: body mass index; CRC: colorectal cancer; CI: confidence interval; kg: kilogram; MMR: mismatch repair; SHR: sub-distribution hazard ratio; m^2: metre square

B. Supplementary Figure

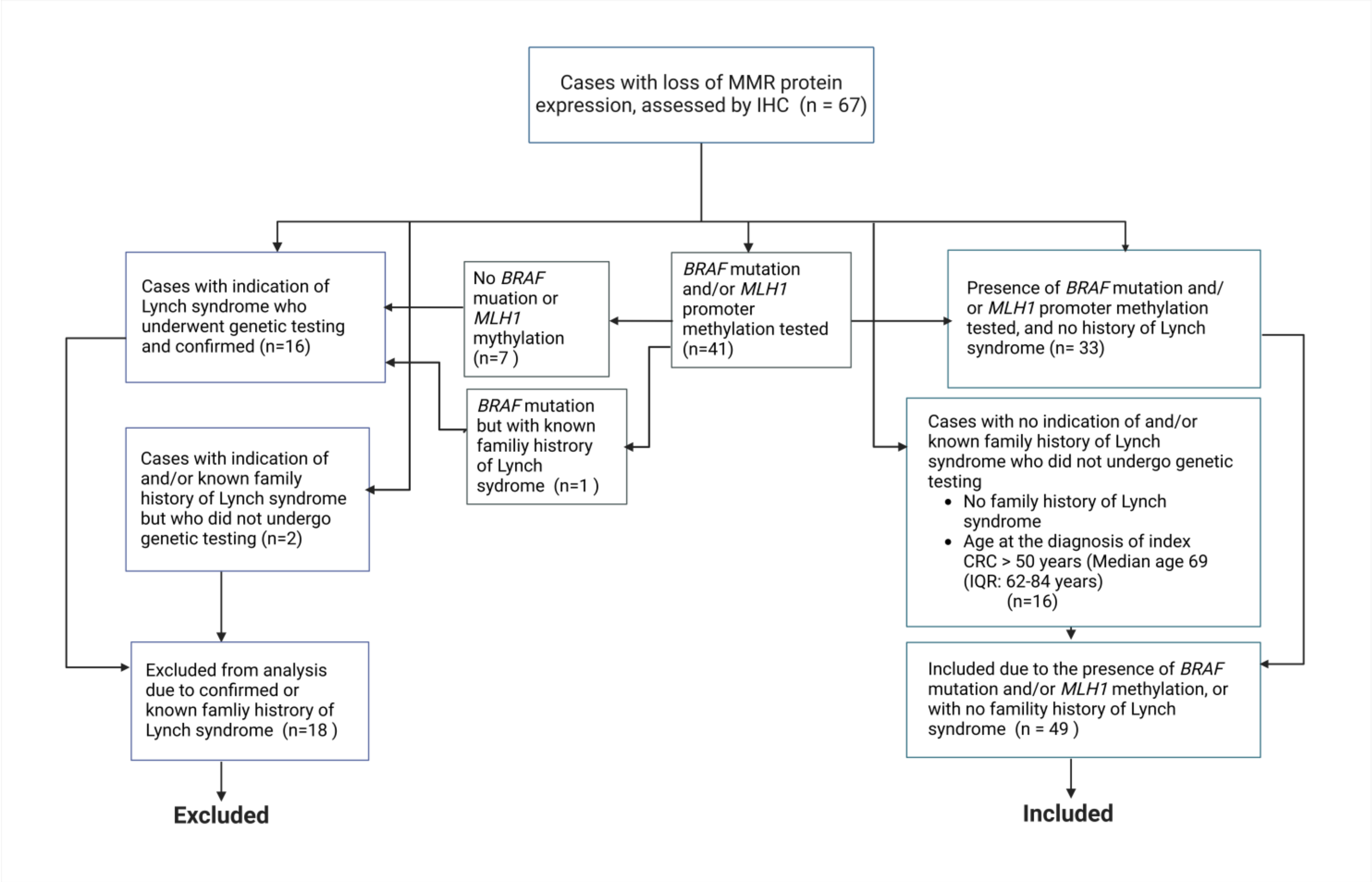

Supplementary Figure S1: Flow chart of excluding confirmed cases or those with a known family history of Lynch syndrome from the study. CRC: colorectal cancer; IHC: immunohistochemistry; IQR: inter quartile range; MMR: mismatch repair
